# Saturnism from Projectiles

**Published:** 1916-10

**Authors:** 


**Saturnism From Projectiles.** Loeper and G. Verpy, Le Progres Med., June 5, obtained the lead reaction in the urine of 6 out of 16 soldiers carrying lead bullets of one kind or another. ½-1 m.g. was found per liter, the estimate being made that a soldier carrying a bullet of 20 grams would eliminate 1½ m.g. of lead a day. In 38 cases examined, albuminuria occurred in 4, the amount varying from ½ to 3 grams a day. Casts were present at times. 2 cases showed marked hypertension, 210 and 220 m.m. 2 showed marked anaemia, 2,800,000 and 3,200,000. Thus while uncommon and often detected with difficulty, they consider that lead poisoning may occur from this cause.

